# Original bundle-based management of septic shock in nagoya university emergency icu

**DOI:** 10.1186/2197-425X-3-S1-A228

**Published:** 2015-10-01

**Authors:** N Matsuda, S Matsushima, H Umino, T Higashi, G Makishi, T Yoshida, Y Shioya, A Numaguchi

**Affiliations:** Emergency & Critical Care Medicine, Nagoya University Graduate School of Medicine, Nagoya, Japan

## Intr

Nagoya University Hospital set up an original management bundle for septic shock on 1st May 2011. This study was intended to verify the strategy.

## Methods

This study adopted retrospective analysis. As the basis for management of septic shock, 13 main elements were combined as the management bundle of septic shock, which contained antibiotics followed culture sampling, standard precaution, infusion therapy adjusted with echocardiogram, early goal-directed original infusion methods, lactate clearance, open lung strategy, analgesia and sedation, β-adrenergic receptor non-stimulation, urine volume management, continuous henofiltration, early enteral nutrition within 48 hours, and early rehabilitation within three days. We analyzed the feasibility and the outcome for septic shock in accordance with this policy from 1st May 2011 to 31st December 2014 as compared with in-ICU mortality rate of more than 25% in 2010.

## Results

Out of total 1,714 cases managed in our ICU among the period, 96 were included with septic shock. The sex ratio was 63:33, mean age was 64.6 ± 18.7 years old, mean ICU stay was 11.6 ± 13.4 days and APACHE II score was 28.6 ± 7.8. The shock withdrawal rate was 99.0%, and in-ICU mortality and 28 day mortality was 5.2% (n = 5) and 6.3%(n = 6), respectively. The dominant causes of death were DNR order with intra-abdominal infection, intestinal necrosis and soft tissue infection.

## Conclusions

A high survival rate was obtained with our septic shock management bundle as compared to our management in 2010 and sepsis registry in the Japanese Society of Intensive Care Medicine.

## Grant Acknowledgment

Grants-in-Aid for Scientific Research in Japan.

